# Serial Assessment of Immune Status by Circulating CD8^+^ Effector T Cell Frequencies for Posttransplant Infectious Complications

**DOI:** 10.1155/2008/718386

**Published:** 2008-04-29

**Authors:** Shinji Uemoto, Kazue Ozawa, Hiroto Egawa, Yasutsugu Takada, Hiroshi Sato, Satoshi Teramukai, Mureo Kasahara, Kohei Ogawa, Masako Ono, Kenji Takai, Masanori Fukushima, Kayo Inaba, Koichi Tanaka

**Affiliations:** ^1^Department of Transplantation and Immunology, Graduate School of Medicine, Kyoto University, Kyoto 606-8501, Japan; ^2^Hepatic Disease Research Institute, Kyoto 605-0981, Japan; ^3^Division of Bioscience, Shiga University of Medical Science, Shiga 520-2192, Japan; ^4^Division of Clinical Trial Design and Management, Translational Research Center, Kyoto University Hospital, Kyoto 606-8507, Japan; ^5^Division of Cellular Immunology, SRL Inc., Tokyo 192-8535, Japan; ^6^Division of Systemic Life Science, Graduate School of Medicine, Kyoto University, Kyoto 606-8501, Japan; ^7^Institute of Biomedical Research and Innovation, Hyogo 650-0047, Japan

## Abstract

To clarify the role of CD8^+^ effector T cells for infectious complications, 92 recipients were classified according to the hierarchical clustering of preoperative CD8^+^CD45 isoforms: Group I was naive, Group II was effector memory, and Group III was effector (E) T cell-dominant. The posttransplant infection rates progressively increased from 29% in Group I to 64.3% in Group III recipients. The posttransplant immune status was compared with the pretransplant status, based on the measure (% difference) and its graphical form (scatter plot). In Groups I and II, both approaches showed a strong upward deviation from pretransplant status upon posttransplant infection, indicating an enhanced clearance of pathogens. In Group III, in contrast, both approaches showed a clear downward deviation from preoperative status, indicating deficient cytotoxicity. The % E difference and scatter plot can be used as a useful indicator of a posttransplant infectious complication.

## 1. INTRODUCTION

Pre-existing high numbers of memory CD8^+^ T cells represent a potent barrier for tolerance induction and affect the course of infection [1, 2]. Our previous findings accord this, since recipients with pre-existing enriched effector T cells (subset CD45RO^−^CCR7^−^) had a high incidence of posttransplant infection and poor survival probability [3]. We, therefore, examined whether qualitative changes in CD8^+^ effector T cell responses to viral or bacterial antigens could explain why these pathogens are not eliminated in most recipients with pre-existing high numbers of effector T cells. Indeed, posttransplant immune status remarkably differs among heterogeneous recipients, particularly at various times after transplantation. Posttransplant immune alloreactive responses are highly complicated by immunosuppression, rejection, and infection. Although the role of immunosuppressive drugs is to inhibit the alloimmune response, they render transplanted recipients highly immunodeficient and susceptible to bacterial and viral infections. Accordingly, we are interested in how the cytotoxic T cell generation changes in response to posttransplant infection after administration of immunosuppressive drugs in heterogeneous recipients.

The reduction of immunosuppression is a major objective for every transplanted recipient in order to reduce drug side effects and restore immunity against common infectious agents. Several strategies show promise, and the predictive values of helper [4], and cytotoxic precursor frequencies [5, 6] in determining graft outcome. Measurements of cytotoxic precursor frequencies have been used for immune monitoring in an attempt to identify recipients suitable for immunosuppression tapering [7] or withdrawal [8]. Other approaches such as “trans vivo” assays [9, 10], interferon-gamma (IFN-*γ*) [11] or IL-2 [12] secretion assays have also been applied. Major histocompatibility complex class I tetramer technology has recently proved clinically useful in the monitoring of immunity to infectious diseases caused by different viruses. However, the use of human leukocyte antigens (HLA) tetramer in a transplant setting is inadequate since it is routinely available only for a limited number of recipients due to the heterogeneity of allopeptides and allogenic HLA molecules. Our study aims to clarify the phenotypic and functional changes in the CD8^+^ subpopulation in many recipients with or without viral infection. We, therefore, used phenotypic analyses for CD8^+^ and CD4^+^ T cells to classify them into effector, effector memory (EM), central memory (CM), and naive cells. We believe that conventional methods of this sort are fully adequate to follow up the clinical outcome in a longitudinal study and to make inferences regarding the clinical situation.

A number of above and other assay [13, 14] are presently being evaluated for efficacy in posttransplant immune monitoring. However, the time-consuming assays with labor-intensiveness or the expensive cost-consuming nature of these assays have prevented their broad acceptance as reliable immune monitoring tools. Since posttransplant infectious complications are the greatest factor leading to seriously poor outcomes, we need simple and effective assays of current immune status for predicting such complications that can be applied to a large cohort of transplanted recipients.

We postulated that immune ability would always recover to preoperative levels, even though it is transiently down-regulated in the early postoperative period. Hence, we investigated how posttransplant immune status evaluated by circulating effector T cells deviates from pretransplant status at various times after living donor liver transplantation (LDLT) and leads to severe infectious complications.

## 2. PATIENTS AND METHODS

### 2.1. Patients and grafts

We examined 92 recipients who had undergone standard LDLT between 2002 and 2006 at Kyoto University Hospital. The ABO incompatible recipients were excluded from this study. Written informed consent was obtained from the recipients before starting the study, which was approved by the Ethics Committee of Kyoto University Hospital and conducted in accordance with the 1975 Declaration of Helsinki, as revised in 1996.

### 2.2. Immunosuppression

Methylprednisolone (initial steroid bolus, ISB; 10 mg/kg) was administered just prior to the start of graft reperfusion. Afterward, two types of immunosuppression protocol in this study were routinely applied: (1) a regular protocol using tacrolimus (Tac) and corticosteroid; and (2) steroid-free protocol using Tac and mycophenolate mofetil instead of corticosteroid for hepatitis C virus (HCV) patients. The regular protocol was followed in 85 (92.4%) of 92 recipients, and the steroid-free protocol in the remaining 7 (7.6%) HCV-infected recipients. The recipients receiving the steroid-free protocol comprised 3 in Group I, 1 in Group II, and 3 in Group III. The doses and timing of those immunosuppressive agents were described in more details in the previous two papers [3, 15].

### 2.3. Definition of an infectious complication

A bacterial, viral, or fungal infection was assumed to have developed if clinical and/or laboratory evidence consistent with acute infection developed. Such laboratory evidence included relevant positive serologic markers and cultures [16]. The criteria for sepsis defined by Bone were applied [17].

### 2.4. Virology

Serum HCV-ribonucleic acid was determined qualitatively by applying the polymerase chain reaction according to the protocols provided with a commercially available assay (Amplicor HCV; Roche Molecular Systems, Pleasanton, Calif, USA).

### 2.5. Tissue typing

Serologic tissue typing for HLA-A, B (Bw), C, DR, and DQ for class I and II loci was undertaken in all recipients.

### 2.6. Flow cytometry

We used peripheral blood mononuclear cells (PBMCs) in all recipients. Sample analysis was always performed within 24 hours after sampling. Since the numbers of CD8^+^ T cells decreased often postoperatively to lower than 10% of lymphocytes, we had to analyze CD8^+^ T cell subsets with low numbers of events, and consequently always performed at least duplicate assays of the same sample. Cell staining was undertaken using monoclonal antibodies as previously reported [3]. The monoclonal antibodies used to stain cell surface antigens were as follows: allophycocyanin (Coulter Immunotech, Miami, FL, USA) or PC-5 (Coulter Immunotech, Marseilles, France)-conjugated anti-CD4 or CD8, fluorescein isothiocyanate-conjugated anti-CD45RO (Nichirei, Tokyo, Japan), TC-conjugated anti-CD45RA (Caltag Laboratories, Burlingame, Calif, USA), phycoerythrin-conjugated anti-CD3 (Coulter Immunotech, Miami, FL, USA), fluorescein isothiocyanate-conjugated anti-CD19 (Coulter Immunotech, Marseilles, France), phycoerythrin-conjugated anti-human CCR7 (DakoCytomation, Kyoto, Japan), phycoerythrin-conjugated anti-CD27 (Coulter Immunotech, Marseilles, France), and fluorescein isothiocyanate-conjugated anti-CD28 (Nichirei, Tokyo, Japan).

#### 2.6.1. Flow cytometric detection of cytokine production and intracellular staining for perforin

Flow cytometric measurement of cytokine production was performed as described previously [3]. Cells were stimulated with a mixture of PMA (25 ng/mL, Sigma-Aldrich Chemical Co., MO, USA) and ionomycin (1 *μ*g/mL, Sigma-Aldrich) with the Golgi inhibitor brefeldin A (10 *μ*g/mL, Sigma-Aldrich). We measured intracellular perforin in CD8^+^ cells without previous stimulation. The perforin analysis was performed according to the previously reported method [3, 18].

### 2.7. Discrimination of first phase (1st) and second phase (2nd) after LDLT

The posttransplant period after LDLT was divided into two phases. The 1st phase is the 24-hour period immediately after graft reperfusion (before Tac administration) and the 2nd phase is the period after the administration of Tac and other immunosuppressants. The upregulation of effector T cells in the 1st phase is mainly due to a vigorous alloreactive response to alloantigen from the donor graft, whereas that in the 2nd phase occurs predominantly as a result of infectious agents.

### 2.8. Statistical analysis

**Figure 1:**
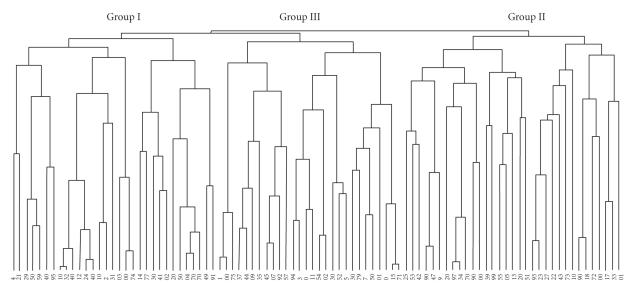
Dendrogram of hierarchical clustering.

We undertook a hierarchical cluster analysis [19] using JMP 5 (SAS Institute Inc., Cary, NC, USA) to identify clusters of recipients having similar distributions of naive-, CM-, EM-, and effector T cells. Hierarchical clustering produced a series of partitions of the data. The first consists of single-patient clusters, and the last consists of a single group containing all individuals. At each stage, the methods fuse groups of individuals, which are most similar. We chose three as an optimal number of clusters based on subjective expertise, as shown in the dendrogram of Figure 1.

The posttransplant immune status was evaluated according to the following measure and its graphical form.

#### 2.8.1. % difference as the measure

To quantify changes in posttransplant alloreactive responses, the proportion of CD8^+^ T cell subsets immediately before LDLT (pretransplant immune status) was subtracted from the proportion at various times after LDLT and is expressed as % difference. This value reflects current immune status after LDLT. Similarly, the % difference was calculated for other variables such as IFN-*γ*, perforin, and CD27^−^CD28^−^ subsets. By this assay, it is possible to compare posttransplant immune status between heterogeneous recipients.

#### 2.8.2. Scatter plot as the graphical form

The deviation points of the CD8^+^ E difference (*Y* axis) from pretransplant values were plotted as a function of the proportion of pre-existing CD8^+^ effector T cells (*X* axis) for each recipient. The 0 point in the *Y* axis indicates immune status just before LDLT. The slope of the correlation line was unity from the definition in all heterogeneous recipients.

The relation between continuous variables was investigated by means of Pearson’s correlation coefficient. Comparisons for continuous variables between groups were undertaken by applying Student’s *t*-test and analysis of variance. Comparisons for proportions between groups were undertaken using Fisher’s exact test or chi-square test. All statistical tests were 2-tailed. Statistical significance was defined as *P* < .05.

## 3. RESULTS

### 3.1. Hierarchical clustering according to preoperative CD8^**+**^CD45 isoform profiles

The ABO-identical and -compatible recipients (*n* = 92) were divided into three groups according to hierarchical clustering based on pretransplant CD3^+^CD8^+^CD45 isoforms (Table 1(a)). The median age of all the recipients was 53 years (range: 19 to 67 years). The age of Group I was significantly younger than in Groups II and III. The pretransplant naive T cell population was most abundant in Group I, EM T cells were the most prevalent in Group II, and effector T cells were the most abundant in Group III. Accordingly, Groups I, II, and III were designated as naive−, EM−, and effector-cell dominant, respectively. The proportion of effector T cell increased significantly and progressively increased from Group I to Group III (Table 1(b)). The CM T cell proportion was significantly higher in Group II than in Groups I and III.

Table 1(c) shows profiles of the recipients and the surgery. One Group II recipient had both hepatitis B virus (HBV) and HCV infection and was classified into both the HBV and HCV groups, so in total 63 recipients were infected with HBV (*n* = 24) or HCV (*n* = 40), while 29 were not infected with either virus (nonviral). The primary diseases of the nonviral recipients were biliary atresia (*n* = 3), primary biliary cirrhosis (*n* = 10), fulminant hepatic failure (*n* = 6), primary sclerosing cholangitis (*n* = 3), alcoholic liver cirrhosis (*n* = 2), liver cirrhosis (unclear) (*n* = 1), polycystic disease (*n* = 1), Caroli disease (*n* = 1), hepatocellular carcinoma (*n* = 1), and autoimmune hepatitis (*n* = 1). The clinical status of the 3 groups did not significantly differ according to the model for end-stage liver disease score [20] and operation profiles. The HLA mismatched loci (>3) were statistically high in Group III rather than in Groups I and II. The possibility that a high number of HLA mismatches may be, at least partly, related to the development of posttransplant infection, cannot be denied. In the pretransplant Cytomegalovirus (CMV) and EBV statuses, CMV-positive recipients were 90% of Group I, 81% of Group II, and 88% of Group III; EBV-positive recipients were 83% of Group I, 93% of Group II, and 85% of Group III. In donors, 71% of Group I, 91% of Group II, and 92% of Group III were CMV positive, and 64% of Group I, 81% of Group II, and 58% of Group III were EBV positive. There was no significant difference in the frequency. The frequencies of donor-positive and recipient-negative statuses were also similar among the three groups, as shown in Table 1(c). Also, there was not different in the operation profiles 3 groups.

### 3.2. Changes in the **%** difference of CD8^**+**^ T cell subsets and the scatter plot after LDLT in 3 groups

**Figure 2:**
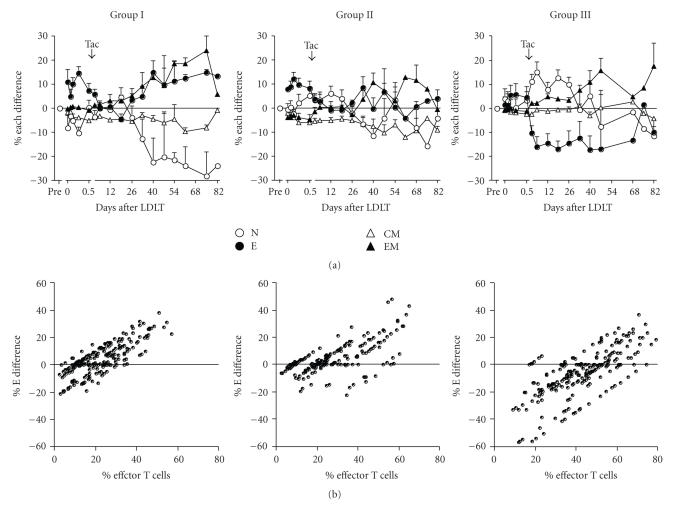
Changes in the % difference of the CD8^+^ T cell subsets (a) and the scatter plot (b) after LDLT in 31 Group I, 33 Group II, and 28 Group III recipients. The % difference was expressed by subtracting the proportion of CD8^+^ T cell subsets immediately before LDLT from the proportion at various times after LDLT. In the scatter plot, the deviation points of the % E difference from pretransplant values were plotted as a function of the proportion of pre-existing CD8^+^ effector T cells for each recipient. N, naive T cells; E, effector T cells; CM, central memory T cells; EM, effector memory T cells; Tac, tacrolimus.

Figure 2(a) shows the additional changes of CD8^+^ T cell subsets burden by LDLT in comparison with the pretransplant immune status, which is expressed as the % difference, after LDLT in 3 groups. In 3 groups, the % E difference was promptly upregulated maximally at 6 hours after graft reperfusion and then decreased to near or below the pretransplant levels after Tac administration. The maximum levels of upregulation at 6 hours were considerably higher in Groups I and II than in Group III. In Group I, after Tac administration the % E difference increased from day 26 to the maximum at day 40 and then remained at the same levels throughout the posttransplant period, accompanied by similar upregulation of % EM difference. The % naive difference correlated significantly (*r* = −0.741, *P* < .0001) negatively with the % E difference. The % CM difference remained unchanged after LDLT. In Group II, the % E difference increased to several % at day 30 but remained at near the baseline in the other period, showing negative (*r* = −0.559, *P* < .0001) % difference of naive and effector T cells. The % CM difference remained unchanged after LDLT. By contrast, in Group III the % E difference was greatly downregulated to far below pretransplant levels and then remained at the same levels for a prolonged period. The % EM difference was increased to approximately 10% over the pretransplant levels at day 50. The % naive difference significantly (*r* = −0.803, *P* < .0001) correlated negatively with % E difference. The % CM difference remained unchanged around the baseline.

Figure 2(b) shows the scatter plot points for the posttransplant CD8^+^ % E difference in relation to the pretransplant proportion of % effector T cells for each of the heterogeneous recipients. The range of the % CD8^+^ effector T cells on the baseline, equivalent to pre-existing values before LDLT, progressively increased from Group I, through Group II, to Group III recipients. In Groups I and II, the scatter plot shows a clear left shift together with many upregulation points and few downregulation points of % E difference from the baseline. In Group III, in sharp contrast, the scatter plot shows a clear right shift together with significantly many downregulation points and few upregulation points from the baseline. In addition, the greater the right shift, the more the % E difference was downregulated. More importantly, the deviation of the % E difference was relatively similar in Groups I and II, although pre-existing effector T cell proportion was slightly higher in Group II than in Group I. In contrast, in Group III the deviation of % E difference was greatly downregulated while the pre-existing effector T cells were the highest.

From the results mentioned above, it is likely that in Groups I and II the CD8^+^ T cells have the full capacity to efficiently induce cytotoxic activity in response to invading pathogen (so-called hyperresponsive), as evidenced by upregulation of effector T cells and IFN-*γ* expression. By contrast, in Group III the CD8^+^ T cells cannot induce cytotoxic T lymphocyte (CTL) cytotoxicity by their downregulation (hyporesponsive or exhausted). It seems likely that immune status after LDLT is determined by pre-existing levels of effector T cells.

### 3.3. Longitudinally prolonged follow-up of the **%** E difference and scatter plot in Group III recipient

**Figure 3:**
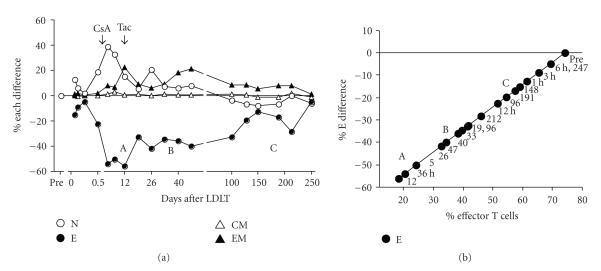
Changes in % difference in CD8^+^ naive, CM, EM, and effector T cells after LDLT in a 55-year-old female recipient. Right, scatter plot between % E difference and % effector T cell proportion. A, period associated with acute cellular rejection, infection and liver damage. B, period associated with acute cellular rejection and aspartate aminotransferase elevation. C, aspartate aminotransferase elevation. White circles denote naive (N), black circles denote effector (E), white triangles denote central memory (CM), black triangles denote effector memory (EM), Pre, pre-LDLT; CsA, cyclosporine A; Tac, tacrolimus; the number below black circles in right figure, postoperative days.

Figure 3 shows the additional changes in the % difference of CD8^+^ T cell subsets burden by LDLT in comparison with the pretransplant immune status at various times in a 55-year-old female recipient who underwent LDLT to treat HCV-related liver cirrhosis and hepatocellular carcinoma. Her extant CD8^+^ naive T cell proportion was only 13.3% (Group III). The CD8^+^ % E difference was significantly downregulated after cyclosporine administration and then remained at the lowest levels far from the baseline until day 220 (left). By day 12, this patient developed acute cellular rejection, infection, and aspartate aminotransferase was elevated (“A” zone of lowest levels). Between days 25–50, acute cellular rejection and elevated serum transaminases persisted (“B” zone). The CD8^+^ % E difference gradually increased at day 150. Around day 200, the % E difference transiently downregulated with highly elevated aspartate aminotransferase, and then the patient was discharged on day 247 when the % E difference reached baseline. The % differences of EM T cells in CD8^+^ T cells were always above baseline. The scatter plot (right) shows that the % E difference progressively increased from zone A to zone C in her clinical course.

### 3.4. Frequencies of posttransplant infection, rejection, life-threatening infectious complication (LTC), and hospital mortality after LDLT in 3 groups

Table 2 shows the frequencies of posttransplant infection, rejection, LTC, and hospital mortality after LDLT in 3 groups of nonviral-, HCV-, and HCV-related recipients.

#### 3.4.1. Infection

The postoperative infection rates progressively increased from Group I to III recipients. The bacterial infection rate progressively increased from Group I to III recipients. The frequencies of infection were significantly higher in Group III than in Groups I and II. Bacterial infections were caused mostly by *Staphylococci, Enterococci,* or *Pseudomonas* species. The fungal infection rates were the highest (21.4%) in Group III than in Groups I (6.5%) or II (6.1%). Moreover, logistic regression analysis adjusted for age, gender, and primary disease showed that the frequency of infection was significantly greater in Group III than Group I (odds ratio: 5.69, 95% CI: 1.70–19.1, *P* = .005) or Group II (odds ratio: 3.53, 95% CI: 1.15–10.9, *P* = .028). The frequency of bacterial infection was significantly greater in Group III than Group I (odds ratio: 5.70, 95% CI: 1.41–23.0, *P* = .015). The infection frequencies did not differ among nonviral-, HBV-, and HCV-related recipients. The overall incidence of Cytomegaloviral diseases after LDLT was 30.4% with being more frequent in Group III (46.4%) than in Groups I (25.8%) and II (21.2%). LTC such as septic shock, adult respiratory distress syndrome, and hepatic necrosis occurred in 6 (14.7%) of 92 recipients (2 in nonviral, 1 in HBV, and 4 in HCV). The frequencies of LTC were higher in Group III (10.7%) than in Group I (3.2%) and Group II (6.1%).

#### 3.4.2. Rejection

The frequencies of acute cellular rejection were 23 (25%) of 92 recipients with being more frequent in Group II (33.3%) and Group III (28.6%) than in Group I (12.9%). The frequency of rejection was significantly greater in Group II than Group I (odds ratio: 4.50, 95% CI: 1.15–17.5, *P* = .030).

#### 3.4.3. Hospital mortality rate

The numbers of hospital death were 7 (7.6%) of 92 recipients. The hospital mortality rate was higher in Group II (9.1%) and Group III (10.7%) than in Group I (3.2%).

### 3.5. Changes in posttransplant immune status in representative of Groups I, II, and III

**Figure 4:**
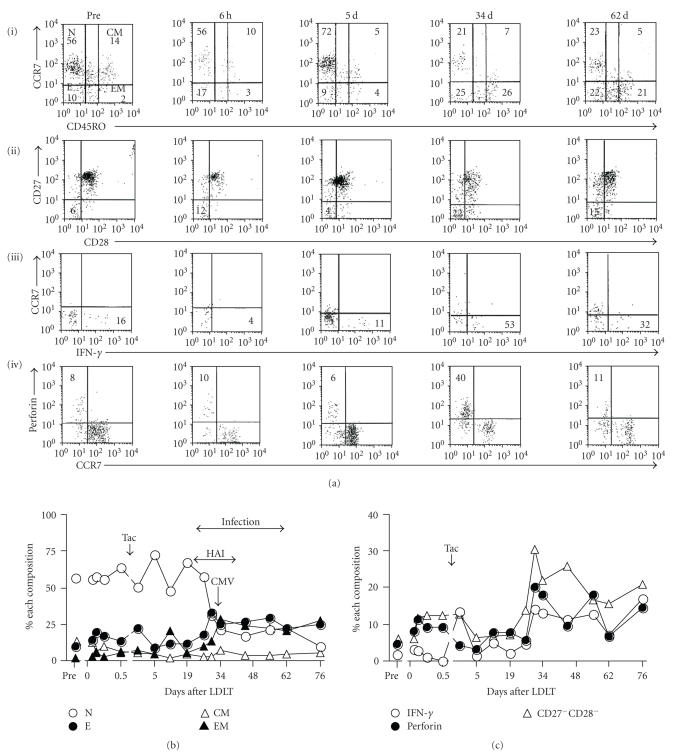
Changes in CD45 isoforms of CD8^+^ T cells, CD27^−^CD28^−^ subsets, IFN-*γ* producing cells and perforin expression after LDLT in a 53-year-old, female recipient who underwent LDLT to treat HCV-related liver cirrhosis. In (a), flow cytometry, using peripheral blood nuclear cells (PBNCs) the lymphocytes were stained with monoclonal antibodies to CD45RO and CCR7. The representative dot plots show double-staining for CD8^+^CCR7/CD45RO on gated lymphocytes (i), which identified 4 subsets of CD8^+^: naive (CD45RO^−^CCR7^+^), central memory (CD45RO^+^CCR7^+^), effector memory (CD45RO^+^CCR7^−^), and effector T cells (CD45RO^−^CCR7^−^). Other dot plots show double-staining for CD27/CD28 on gated CD8^+^ T cells (ii), CCR7/IFN-*γ* on gated CD8^+^CD45RA^+^ cells (iii), perforin/CCR7 on gated CD8^+^CD45RO^−^ cells (iv). Cells in quadrants are presented as ratios (%). Right low (c), proportions of perforin and IFN-*γ* expression are expressed as ratios (%) of CD8^+^ T cells. Pre, pre-LDLT; Tac, tacrolimus. (b) N, naive T cells; E, effector T cells; CM, central memory T cells; EM, effector memory T cells; and HAI, hepatic artery flow interruption. (c) IFN-*γ*, interferon-gamma; and CD27^−^CD28^−^, CD27^−^CD28^−^ subsets.

Figure 4 shows the distribution of CD8^+^ T cell subsets by flow cytometry (a) and changes in the proportion of CD8^+^ T cell subsets (b) as well as IFN-*γ*, perforin, and CD27^−^CD28^−^ subsets (c) after LDLT in a representative of Group I recipient (53-year-old female) undergoing LDLT to treat HCV-related liver cirrhosis. The proportion of effector T cells increased only slightly during 3–6 hours after graft reperfusion and then decreased by Tac administered from postoperative 24 hours. The proportion of EM and effector T cells increased from day 19, peaking at day 30. The proportion of IFN-*γ*, perforin, and CD27^−^CD28^−^ subsets remained relatively unchanged by day 19 and then increased to maximal levels at day 30 (c). On day 27 after LDLT, Doppler ultrasonography detected interrupted flow in the hepatic artery. Because serum asparatate aminotransferase was minimally increased (about 50 IU/L), anticoagulant therapy was applied to the recipient under close observation. During this period, this recipient developed infection, CMV at day 34, *Enterobacter cloacae* in blood at day 28, *Enterococcus* in catheter at day 50, *Staphylococcus maltophilia,* and *Enterococcus faecalis* in pharynx at day 61, Coagulase negative *Staphylococcus* in blood at day 75, and *Candida* in urine at day 61. The histological examination of a liver biopsy specimen performed at day 35 showed pericentral venous hemorrhage. Doppler ultrasonography on day 41 showed that arterial flow had recovered. The % E difference was slightly downregulated on day 61 in parallel with recirculation of the hepatic artery flow and amelioration of infection. These findings indicate that changes in the % E difference are closely related to the incidence of posttransplant infection.

**Figure 5:**
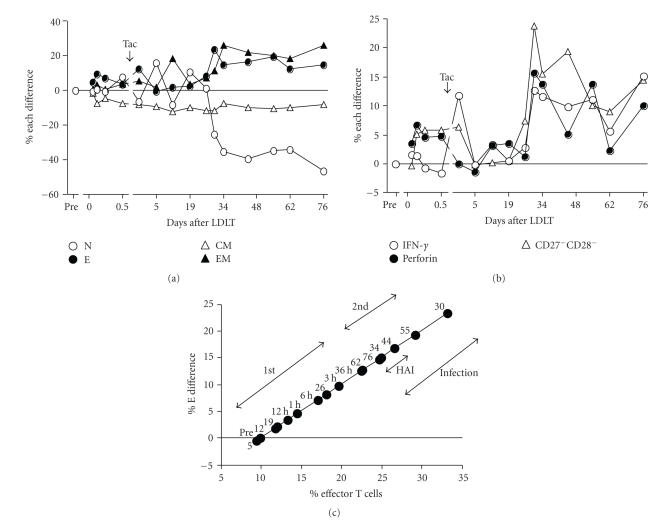
Changes in % difference in CD8^+^ naive, CM, EM, and effector T cells, as well as IFN-*γ*, perforin, and CD27^−^CD28^−^ subsets after LDLT in same recipient shown in Figure 4. Low (c), scatter plot between % E difference and % effector T cell proportion. 1st phase is the period before Tac administration. 2nd phase is the period after Tac administration. Pre, pre-LDLT; Tac, tacrolimus; the number above black circles in (c) figure, postoperative days. (a) N, naive T cells; E, effector T cells; CM, central memory T cells; and EM, effector memory T cells. (b) IFN-*γ*, interferon-gamma; and CD27^−^CD28^−^, CD27^−^CD28^−^ subsets. (c) HAI, hepatic artery flow interruption.

Figure 5 shows the additional changes in the % difference of CD8^+^ T cell subsets as well as IFN-*γ*, perforin, and CD27^−^CD28^−^ subsets burden by LDLT in comparison with the pretransplant immune status at various times in the same recipient of Figure 4. The % E difference increased only slightly during 3–6 hours and then decreased after Tac administration, further increasing again from day 19, along with an increase in % EM difference (a). The % naive difference greatly downregulated from day 30, while the % CM difference remained unchanged at slightly below the baseline throughout the post-LDLT period. On the other hand, the % difference of IFN-*γ*, perforin, and CD27^−^CD28^−^ subsets increased slightly during the 3–12 hours period, decreased to pretransplant levels after Tac administration and then began to increase from day 19, peaking at day 30, simultaneously with the development of infection (b). The low (c) figure shows the scatter plot of posttransplant deviation of the % E difference from the pre-existing effector T cell proportion for this recipient. All points were situated above the baseline. The upregulation of % E difference immediately after graft reperfusion (1st phase) was less than 10% above the baseline. With the onset of severe infection following hepatic artery flow interruption, the scatter plot points were located at the highest and furthest point from zero (2nd phase). These results indicate that the deviation of the % E difference is greatest when there is a vigorous response to infection.

**Figure 6:**
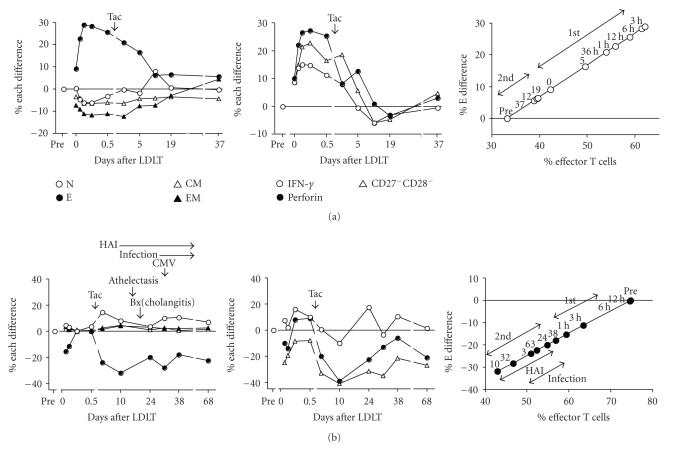
(a) Changes in % difference in CD8^+^ naive, CM, EM, and effector T cells, as well as IFN-*γ*, perforin, and CD27^−^CD28^−^ subsets in a 62-year-old male recipient undergoing LDLT to treat HCV-related liver cirrhosis and hepatocellular carcinoma. Right, scatter plot between % E difference and % effector T cell proportion. 1st phase is the period before Tac administration. 2nd phase is the period after Tac administration. Pre, pre-LDLT; Tac, tacrolimus; the number above black circles in right figure, postoperative days. Left figure: N, naive T cells; E, effector T cells; CM, central memory T cells; and EM, effector memory T cells. Middle figure: IFN-*γ*, interferon-gamma; and CD27^−^CD28^−^, CD27^−^CD28^−^ subsets. (b) Posttransplant changes in % difference in CD8^+^ T cell subsets as well as IFN-*γ*, perforin, and CD27^−^CD28^−^ subsets in a 60-year male recipient undergoing to treat HBV-related liver cirrhosis and hepatocellular carcinoma. Right, Scatter plot between % E difference and % effector T cell proportion. 1st phase is the period before Tac administration. 2nd phase is the period after Tac administration. Pre, pre-LDLT; Tac, tacrolimus; HAI, hepatic artery flow interruption; Bx, biopsy; the number above black circles in right figure, postoperative days.

Figure 6(a) shows additional changes in the % difference of CD8^+^ T cell subsets as well as IFN-*γ*, perforin, and CD27^−^CD28^−^ subsets burden after LDLT in comparison with the pretransplant immune status at various times and the scatter plots showing the relationship between the % E difference and the proportion of CD8^+^ effector T cells in a representative of Group II recipient (62-year-old male) undergoing LDLT to treat HCV-related cirrhosis and hepatocellular carcinoma. The % E difference increased greatly in the 3–6 hours period, and then decreased to the baseline after Tac administration. The % difference of the other subsets remained unchanged at slightly below the baseline throughout (left). The % difference of IFN-*γ*, perforin, and CD27^−^CD28^−^ subsets increased greatly from 3–12 hours but decreased to the baseline after Tac administration (middle). All the scatter plots were above the baseline, showing the highest % difference during the vigorous alloreactive response in the 1–12 hours period after graft reperfusion (1st phase), before returning to near the baseline after Tac administration (2nd phase) (right). This recipient was discharged uneventfully at day 45 without development of infection.

Figure 6(b) shows changes in the % difference in CD8^+^ T cell subsets as well as IFN-*γ*, perforin, and CD27^−^CD28^−^ subsets after LDLT in a representative of Group III recipient (60-year-old male) undergoing LDLT to treat HBV-related cirrhosis and hepatocellular carcinoma. The % E difference decreased profoundly to −30% after Tac administration and remained at this level until day 68. The % naive T cell difference increased slightly, but the other subsets remained at the baseline (left). Corresponding to the decrease of the % E difference, the % difference of IFN-*γ*, perforin, and CD27^−^CD28^−^ subsets was similarly strongly downregulated after Tac administration but increased from day 24 (middle). All scatter plots were situated below the baseline (right), between 0 and –30% in the period immediately after graft reperfusion (1st phase) but showed strong further downregulation also after Tac administration (2nd phase). During operation, the hepatic artery anastomosis was impossible because of the vascular anomaly. A moderate elevation of serum aspartate aminotransferase and C-reactive protein continued from day 19. Athelectasis of the lungs developed at day 15 and infection occurred from day 10; *Enterobacter cloacae* and *Pseudomonas aeruginosa* presented in urine at day 10 and 53; and CMV at day 32. At day 19, the histological examination of liver biopsy specimen showed cholestasis and cholangitis. After discharge at postoperative day 81, this recipient had to be very often hospitalized due to recurrence of severe infection combined with septic shock, possibly secondary to prolonged downregulation of % E difference.

### 3.6. Changes in the **%** IFN-***γ*** difference after LDLT

**Figure 7:**
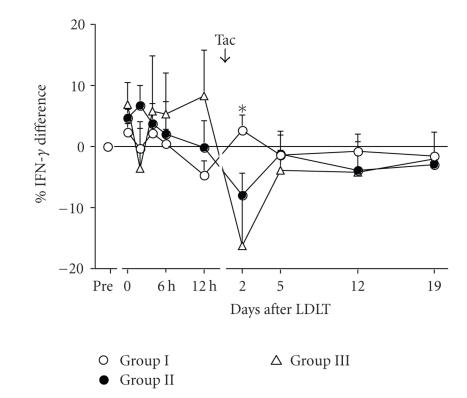
Changes in the % IFN-*γ* difference after LDLT in 3 groups. *P-value at day 2 is based on analysis of variance: all, .0161. *P*-values between 3 groups are based on Student’s *t*-test: Group I versus II, .0248; Group I versus III, .0133; and Group II versus III, .3058.

Figure 7 shows changes in the % IFN-*γ* difference after LDLT in 3 groups. Immediately after graft reperfusion, the % IFN-*γ* difference upregulated at 3–6 hours progressively from Group I to Group III recipients. By contrast, after Tac administration, conversely, the % IFN-*γ* difference downregulated progressively from Group I to III recipients. The period to restore to pretransplant value was the longest in Group III rather than in Groups I and II.

## 4. DISCUSSION

### 4.1. Better evaluation of posttransplant immune status by the **%** difference rather than by the proportion

There were markedly different phenotypes and functions of T cells before and after LDLT among the many heterogeneous recipients. The posttransplant immune status cannot be clearly estimated by up- or downregulation of the proportion of T cell subsets, cytokines, and perforin. From the analysis of our extensive data bank, it has already been found that, in order to compare the posttransplant immune status in many recipients, it is particularly useful to estimate the magnitude of the additional immunological load burden by liver transplantation in comparison with the pretransplant immune status, which is expressed as the % difference. The following issues have been clarified by this assay: (1) posttransplant changes in phenotypic and functional properties of CD4^+^ and CD8^+^ T cells, albeit in a different period, can be almost restored to the pretransplant pattern; therefore, the pretransplant value can be chosen as the starting time point; (2) in order to compare the posttransplant immune status among many recipients, their immune status, even though on a small scale, can be exactly estimated by the changes in the % difference rather than the proportion; and (3) CD8^+^ CTL generation varied markedly after LDLT in many recipients. Evaluation of the % difference was closely related to posttransplant infection and the recipient’s survival probability. From these results, it is possible to compare the enhanced immune status induced after LDLT among heterogeneous recipients.

### 4.2. Distinct immune response after LDLT in 3 groups

Antigen-primed CD8^+^ memory T cells can be distinguished by CCR7 and CD45RA expression into distinct long-lived CCR7^+^CD45RA^−^ “central memory,” CCR7^−^CD45RO^+^ “effector memory,” and CCR7^−^CD45RA^+^ “effector subsets” [21]. In Groups I and II, % difference of CD8^+^ CM or EM T cells remained at baseline or slightly above throughout the posttransplant period. However, the % E difference significantly upregulated after LDLT and new invading pathogens were cleared. In Group III, in sharp contrast, the % E difference after LDLT was remarkably downregulated in Group III and accompanied by obvious decreases in IFN-*γ*-producing cells, perforin expression, and CD27^−^CD28^−^ subsets. In those recipients, pathogens could not efficiently be cleared during downregulation of the % E difference, leading to a high posttransplant incidence of critical infectious complications. These results indicate that the pre-existing memory subset population before LDLT restricted such changes in CD8^+^ memory subsets after LDLT in the 3 groups, indicating the so-called heterologous immunity [1, 2].

A potentially important factor contributing to posttransplant infectious complication is the inability of CD8^+^ effector T cells to control infection. The clearance of viruses, bacteria, and fungus by the immune response is thought to require the destruction of infected cells by CTLs via perforin. The perforin pathway involves granule exocytosis and might be the most effective in vivo, at least, in terms of controlling microbial infections [22, 23]. The memory CD8^+^ T cells in Groups I and II could acquire cytotoxic activity very rapidly and may have significantly contributed to even the earliest control of viral replication and bacteria by killing infected cells. Thereafter, lytic activity would have gradually decreased as the infection was cleared, as previously reported [24]. In contrast, CTL cytotoxicity brought about by perforin expression was greatly impaired in severely infected Group III recipients. A lag time may exist between the downregulation of % E difference and the restoration of % E difference associated with the ability to clear these invading pathogens. If the great downregulation continued for a prolonged period, it rendered transplanted recipients highly susceptible to infection and was refractory to pathogen clearance. Viral and bacterial pathogens in those recipients might easily be activated and continuously retained during the prolonged lag time. The clear downregulation of % E difference might be equivalent to the functional exhaustion proposed by Wherry et al [25]. Thus, changes in the % E difference after LDLT can be mainly classified into two groups of CD8^+^ T cells with either a high or a low proportion of pre-existing effector T cells. The alloimmunity of Groups I and II was associated with upregulation of CTL cytotoxicity in response to infection-hyperresponsive, whereas Group III was accompanied by downregulation of cytotoxicity hyporesponsive.

### 4.3. Proposed role of Tac inducing the downregulation of **%** E difference

As factors contributing to downregulation of effector T cells, various factors such as inappropriate immunosuppression, infection, and rejection could be considered. Among them, the Tac administration in Groups I and II suppressed a vigorous immune response against alloantigens from the transplanted allograft immediately after LDLT, as indicated by normalization of the % E difference or scatter plot to around baseline at posttransplant day 2, when the trough reached to an appropriate level. In Group III, in contrast, the % E difference was greatly downregulated after Tac administration and took more prolonged time to restore to pretransplant levels than in Groups I and II. The mechanism is now unknown, but it seems likely that the CD8^+^ T cells enriched with pre-existing high effector T cell may have the highest native impact to Tac administration. On the other hand, in the experimental study, it has been found that in the mice dominated by IFN-*γ* production the carcineurin-inhibitor (Tac and cyclosporine A) therapy effectively induced long-term graft survival after cardiac allografting, whereas in IFN-*γ*-deficient mice the same therapy showed only marginally prolonged graft survival, along with resistance to carcineurin inhibitor [26, 27]. Accordingly, it seems likely that in the effector-dominant recipients the CTL activity cannot effectively respond to Tac therapy during downregulation of IFN-*γ* production.

### 4.4. Posttransplant infection and acute cellular rejection

The interrelationships between immunosuppression, infections, and allograft rejection are certainly highly complex and incompletely understood [28]. In this study, most of the acute cellular rejection occurred in the period of downregulation of the % E difference but not at the time of upregulation. This may be consistent with the facts that the heterologous memory generated by bacterial and viral antigens has been shown to be a barrier for transplantation tolerance and is associated with increased rates of acute rejection and impaired graft function [1, 2].

## 5. CONCLUSION

### 5.1. Novel perspective

The favorable outcome of LDLT is the prompt restoration of the immune system to pretransplant status associated with active immune regulation, as evidenced by maintaining a % E difference or scatter plot to around the baseline. The naive-dominant recipients are uniformly highly sensitive to pathogen clearance. Indeed, their CTLs were generated in response to infection earlier and more rapidly in CD8^+^ naive-dominant, than in either CD8^+^ EM- or effector-dominant T cells [24].

From the results mentioned above, preserving immunity by minimizing immunosuppression might be one of the most important challenges in posttransplant management in Group III. The assay of % E difference or scatter plot is simply practicable and can be used as a potentially useful marker for posttransplant infectious complications. Firstly, the classification of naive-, EM-, and effector-dominant recipients is done according to pre-existing CD8^+^ T cell subsets. Second, if the recipient belongs to Group III, the magnitude of downregulation of % E difference at the posttransplant day 2 after Tac administration is determined. At present, we are now working to determine whether it is possible to tailor immunosuppression for high-risk Group III recipients to improve their clinical outcomes. Such consideration will open the door to novel and better targeted therapies.

## Figures and Tables

**Table 1(a):** 

Group	*n*	Age (*y*)	% Naive	% CM	% EM	% Effector	% Lymphocytes*	% CD4^†^	% CD8^†^
			T cells	T cells	T cells	T cells			
I	31	47 ± 11	53.44 ± 10.42	7.56 ± 4.19	7.01 ± 5.53	17.51 ± 7.76	19.81 ± 10.34	47.05 ± 15.50	20.83 ± 10.26
II	33	53 ± 8	20.80 ± 10.99	11.88 ± 6.04	20.24 ± 7.21	24.25 ± 11.66	21.22 ± 10.24	46.86 ± 11.54	19.71 ± 9.17
III	28	53 ± 12	21.22 ± 10.52	5.88 ± 3.43	7.33 ± 4.59	48.36 ± 12.77	20.76 ± 11.56	42.89 ± 12.75	21.74 ± 7.47

**Table 1(b):** 

Variance
All	*P* ^‡^	.0501	<.0001	<.0001	<.0001	<.0001	.8693	.4087	.6845
I versus II	*P* ^‡^	.0223	<.0001	.0016	<.0001	.0089	.5910	.9567	.6480
I versus III	*P* ^‡^	.0647	<.0001	.1001	.9287	<.0001	.7446	.2681	.7004
II versus III	*P* ^‡^	.9133	.8840	<.0001	<.0001	<.0001	.8735	.2066	.3534

**Table 1(c):** 

		Group I	Group II	Group III	*P*
Recipient (male/female)		31 (18/13)	33 (23/10)	28 (15/13)	.4049^‡^
Original liver disease		—	—	—	.1456^‡^
Nonviral		14	9	6	—
HBV		6	12	6	—
HCV		11	13	16	—
MELD		17 ± 10	16 ± 9	14 ± 7	.5460^§^
HLA mismatch (0–2/>3)		17/13	16/15	7/21	.0347^‡^
CMV status: R+ (D+/R−)		26/29 (3/29)	25/31 (6/31)	23/26 (2/26)	.5504 (0.3971)^‡^
EBV status: R+ (D+/R−)		24/29 (3/29)	28/30 (2/30)	23/27 (3/27)	.4442 (0.8232)^‡^
Operation profile
GWBR		1.13 ± 0.26	1.33 ± 1.07	1.16 ± 0.27	.4422^§^
Ischemic time (min)	Cold	104 ± 74	115 ± 62	144 ± 92	.1276^§^
	Warm	51 ± 24	60 ± 48	54 ± 17	.5502^§^
Blood loss (g)		7647 ± 12227	7761 ± 10135	9084 ± 11086	.8615^§^
Blood loss/BW		0.14 ± 0.28	0.13 ± 0.17	0.15 ± 0.19	.8719^§^

Viral status: R+; recipient with preoperative positive serology, D+/R−; donor-positive and recipient-negative status, *% of peripheral blood mononuclear cells; ^†^% of lymphocytes; ^‡^Categorical variables were compared using Fisher’s exact test or chi-squared test; ^§^Continuous variables between groups were compared using Student’s *t*-test or ANOVA. Values are expressed as mean *±*SD.

**Table 2:** Frequencies of Posttransplant Infection, Complication and Mortality in 3 Groups.

Group	Primary disease	Total	Infection				Rejection	LTC*	Hospital death
				Bacteria	Virus	Fungus
		*n*	*n* (%)	*n* (%)	*n* (%)	*n* (%)	*n* (%)	*n* (%)	*n* (%)
I	Nonviral	14	5	2	5	2	4	1	1
	HBV	6	0	0	0	0	0	0	0
	HCV	11	4	2	3	0	0	0	0
	Total	31	9 (29.0)	4 (12.9)	8 (25.8)	2 (6.5)	4 (12.9)	1 (3.2)	1 (3.2)

II	Nonviral	9	5	4	2	0	2	0	0
	HBV	12^†^	3^†^	3^†^	2^†^	0	5	1^†^	1^†^
	HCV	13^†^	4^†^	3^†^	4^†^	2	4	2^†^	3^†^
	Total	33	11 (33.3)	9 (27.3)	7 (21.2)	2 (6.1)	11 (33.3)	2 (6.1)	3 (9.1)

III	Nonviral	6	5	4	5	3	2	1	1
	HBV	6	4	2	3	0	1	0	0
	HCV	16	9	5	5	3	5	2	2
	Total	28	18 (64.3)	11(39.3)	13 (46.4)	6 (21.4)	8 (28.6)	3 (10.7)	3 (10.7)

All	*P* ^‡^	—	.0117	.0690	.0812	.0985	.1472	.5038	.5131
I versus II	*P* ^§^	—	.7908	.2168	.7711	>.999	.0773	>.999	.6136
I versus III	*P* ^§^	—	.0092	.0346	.1124	.1337	.1974	.3373	.3373
II versus III	*P* ^§^	—	.0215	.4141	.0533	.1272	.7847	.6533	>.999

*LTC, Life-threatening infectious complication; ^†^one recipient had HBV- and HCV-infection; ^‡^ *P*-values are based on chi-square test; ^§^ *P*-values are based on Fisher’s exact test.
